# CT-Based Deep Learning Model for Invasiveness Classification and Micropapillary Pattern Prediction Within Lung Adenocarcinoma

**DOI:** 10.3389/fonc.2020.01186

**Published:** 2020-07-22

**Authors:** Hanlin Ding, Wenjie Xia, Lei Zhang, Qixing Mao, Bowen Cao, Yihang Zhao, Lin Xu, Feng Jiang, Gaochao Dong

**Affiliations:** ^1^Jiangsu Cancer Hospital, Jiangsu Institute of Cancer Research, The Affiliated Cancer Hospital of Nanjing Medical University, Nanjing, China; ^2^Thoracic Surgery Department of Jiangsu Cancer Hospital, Nanjing, China; ^3^Jiangsu Key Laboratory of Molecular and Translational Cancer Research, Cancer Institute of Jiangsu Province, Nanjing, China; ^4^The Fourth Clinical College of Nanjing Medical University, Nanjing, China; ^5^CT/MRI Department of Jiangsu Cancer Hospital, Nanjing, China; ^6^The First Clinical Medical College of Nanjing Medical University, Nanjing, China

**Keywords:** lung adenocarcinoma, micropapillary component, computed tomography, deep learning, convolutional neural network, artificial intelligence

## Abstract

**Objective:** Identification of tumor invasiveness of pulmonary adenocarcinomas before surgery is one of the most important guides to surgical planning. Additionally, preoperative diagnosis of lung adenocarcinoma with micropapillary patterns is also critical for clinical decision making. We aimed to evaluate the accuracy of deep learning models on classifying invasiveness degree and attempted to predict the micropapillary pattern in lung adenocarcinoma.

**Methods:** The records of 291 histopathologically confirmed lung adenocarcinoma patients were retrospectively analyzed and consisted of 61 adenocarcinoma *in situ*, 80 minimally invasive adenocarcinoma, 117 invasive adenocarcinoma, and 33 invasive adenocarcinoma with micropapillary components (>5%). We constructed two diagnostic models, the Lung-DL model and the Dense model, based on the LeNet and the DenseNet architecture, respectively.

**Results:** For distinguishing the nodule invasiveness degree, the area under the curve (AUC) value of the diagnosis with the Lung-DL model is 0.88 and that with the Dense model is 0.86. In the prediction of the micropapillary pattern, overall accuracies of 92 and 72.91% were obtained for the Lung-DL model and the Dense model, respectively.

**Conclusion:** Deep learning was successfully used for the invasiveness classification of pulmonary adenocarcinomas. This is also the first time that deep learning techniques have been used to predict micropapillary patterns. Both tasks can increase efficiency and assist in the creation of precise individualized treatment plans.

## Introduction

Lung cancer is one of the most common cancer incidents worldwide, comprising one-third to one-half of incidents being attributed to adenocarcinoma (1). In 2011, adenocarcinomas were newly classified as adenocarcinoma *in situ* (AIS), minimally invasive adenocarcinoma (MIA), and invasive adenocarcinoma (IA) (2). The micropapillary pattern was added as a new histologic subtype of IA, with the other four currently existing subtypes being lepidic, acinar, papillary, and solid patterns (2). The prognosis of MIA and AIS is quite different from that of IA, and among IA it was demonstrated that the micropapillary-predominant lung adenocarcinoma (MPs) have a more adverse outcome when compared with other subtypes.

Surgical resection is one of the main treatment choices for the early-stage lung adenocarcinomas which are generally recognized as lung nodules on the computed tomography (CT). The resection range depends on the pathological features of the nodule, and surgical plans will differ depending on the prognosis. AIS and MIA are suitable for sublobar resection, with a promising nearly 100% 5-year survival rate. However, for IA, the lobectomy is considered an adequate option given its more optimal surgical outcome than the sublobar resection (3–5). As the disease-free survival at 5 years for MPs is only 67%, a more aggressive extended resection is required consisting of a larger excision area and higher surgical risk (4, 6, 7).

Due to an increased degree of invasiveness with poor prognosis, it is crucial to determine the exact pathological classification of the tumor. An intraoperative frozen section is widely used to distinguish MIA from IA during surgery, and is considered to be the gold standard in clinical practice. Liu et al. illustrated that the total concordance rate between an intraoperative frozen section and the final pathology was 84.4%, and the diagnostic accuracy of the intraoperative frozen section for tumors ≤1 cm in diameter was 79.6% (8). A second operation, which is an unnecessary waste of medical resources, may be required if there is incorrect recognition of the pathological invasiveness stage during surgery. Furthermore, with the exception of the final pathology report after surgery, there are few methods that can recognize MPs before or during resection. Thus, the development of a new, non-invasive method that provides a reference for the invasiveness degree and pathologic subtype before surgery is desired to reduce the occurrence of inappropriate surgical plan choices and optimize the distribution of medical resources.

CT interpretation, as a vital part of modern clinical diagnostic procedures, is critical for the early detection of lung adenocarcinoma, which can reduce lung cancer-specific mortality by 20% (5). The diagnosis and the subsequent treatment of lung adenocarcinoma typically require expert radiologists to analyze the images, depending on the size, morphological feature, or the internal texture of the nodule (9). Many radiologists have attempted to combine the classification task using radiomics with the machine learning technique (6, 10–12). The combination of the radiologic image and the pathologic feature using the artificial intelligence (AI) technique inspired the medical field to develop a new method regarding the processing of medical data, revealing information that otherwise cannot be discovered through the human eye and assessing lesions using a mechanical method. However, when the amount of data becomes huge, the performance did not improve limited by the structure of the model.

Deep learning, as a branch of AI, has emerged due to its unprecedented superior performance in recent image classification competitions. With the use of graphics processing unit (GPU) hardware, the deep learning model can arrange a much larger scale of the dataset and can achieve higher accuracy and stability than the traditional machine learning technique, which has been illustrated in many other fields (13, 14). Deep learning AI can be used as a computer-aided diagnostic system, and can become a part of the clinical diagnostic procedure. It improves the efficiency of the radiologist, saves diagnostic time, and improves diagnostic accuracy. Also, as many researchers have illustrated, deep learning can achieve a better performance than that of many senior medical practitioners addressing tasks (15, 16). Because well-trained and experienced radiologists are not always available in less developed areas, the application of AI can enhance the quality of diagnosis and reduce unnecessary costs during treatment in these locations.

Previous studies have explored the feasibility of using deep learning-assisted analysis of lung nodules, and have achieved promising results. As Nasrullah et al. illustrated (17), deep learning models to classify benign and malignant nodules can reach an accuracy of more than 80%. It has been reported that deep learning in many fields even outperformed senior radiologists (15). However, insights into subtype classification, which cannot be performed by human eyes, remained scarce. We concluded that a deep learning model further focusing on the malignant nodule is required to determine the grade of malignancy and classify the subtype of the nodule.

In our research, we propose the utility of the Convolutional Neural Network (CNN) model to detect the pathologic invasiveness degree of lung nodules on CT scans, and furthermore, attempted to discriminate the IA with MPs from other subtypes. There are two models built in our research, one called the Lung-DL model and the other one was the Dense model. We also compared the performance of different CNN structures. To the best of our knowledge, few researchers have focused on the classification of malignant nodules down to the subtype level using deep learning models (16).

## Materials and Methods

### Creation of Datasets

This research was approved by the Institutional Review Board of Jiangsu Cancer Hospital and Jiangsu Institute of Cancer Research. Due to the retrospective nature of the study, the patient informed consent was waived

For the establishment of the dataset, 1,007 histopathologically confirmed lung cancer patients from Jiangsu Cancer Hospital were originally obtained in our research. First, 19 patients whose pathological stage was atypical hyperplasia and 22 patients diagnosed with squamous carcinoma or other categories were excluded. Among the 966 patients, we excluded patients whose TNM staging is above T_1C_N_0_M_0_. Thus, 25 patients with lymphatic metastasis and 127 patients with more than one nodule were excluded. The remainder of the 814 patients consisted of 72 AIS, 110 MIA, and 35 MPs. Next, we removed corrupted data that cannot successfully open or data with poor resolution. Finally, 61 AIS patients, 80 MIA patients, and 33 MP patients were enrolled.

**Figure 1:**
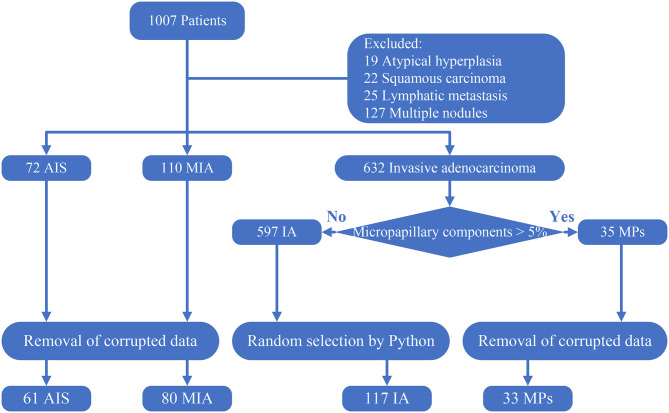
Creation of the dataset. Corrupted data: data that cannot open and data that has a poor resolution. The 117 IA was randomly selected using a Python script from the 597 IA with no micropapillary component.

Because there is a similar prognosis for AIS and MIA, but the prognosis of IA is poorer (3–5), distinguishing IA from AIS and MIA can assist surgeons in planning an operation. However, an imbalance in the amount of data will adversely affect the performance of a deep learning model (18). Therefore, with the original purpose to distinguish IA from AIS and MIA and the consideration to avoid any imbalances in the data amount, a subset of 117 IA without micropapillary was randomly created from the remainder of the 597 IA cases so that the number of total invasive adenocarcinoma (150) was approximately equal to the total amount of MIA and AIS. Finally, a dataset consisting of 61 AIS, 80 MIA, 117 IA, and 33 MPs was constructed. All these processes are illustrated in Figure 1.

In the dataset, 14 AIS, 11 MIA, 20 IA, and 5 MPs were randomly selected to form the test set. A training and validation set was created with the remainder of the dataset, in which 70% of the data (*n* = 169) were randomly selected by the program for training and the other 30% (*n* = 72) for validation of the deep learning model.

### Preprocessing

The CT scans were obtained from the CT/MRI department of Jiangsu Cancer Hospital using a LightSpeed VCT. The scanning matrix was set to 521^*^512 pixels. The slice thickness was 0.625 mm. The reconstructed thickness was 1.25 and 5 mm. The patients enrolled all owned two sets of CT scans with a reconstructed thickness of 1.25 and 5 mm. With the consideration to preserve more vital nodule information required for the research, the 1.25 mm thick CT sets were used for the research, and the 5 mm thick CT sets were abandoned.

Previous studies generally reported a deep learning-based nodule detection accuracy >90% (19). However, insights into subtype classification remained scarce. Therefore, in order to focus on the subtype classification of lung nodules, only 12 slices with the nodule in the center were chosen for labeling. For nodules >13.75 mm in size (that appear in more than 12 slices), the slice at the margin of the nodule was excluded to ensure that most of the information pertaining to the nodule could be preserved.

**Figure 2:**
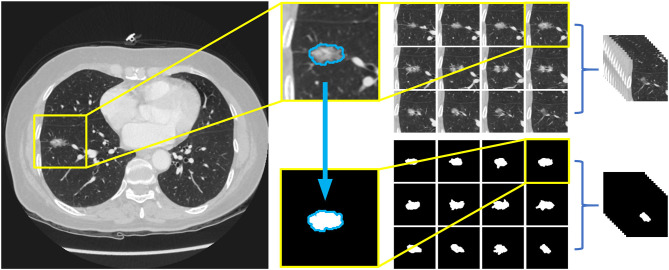
Preprocessing of image data and the arrangement of dataset. Twelve slices chosen for labeling and 12 label files were trimmed to a size of 96*96 pixels with nodules in the middle, and then saved as Dicom format in separate directories, both named with the patients' id.

For the pre-processing of the images, the Amira 6.0.1 software was used to label the nodules in the images. We applied a window range between −1,000 and 400 to assess the images. Then, the images were manually labeled by two investigators (HD and YZ) who were blind to the histological results and reviewed by an experienced radiologist (LZ with 10 years of experience in chest CT diagnosis). The borders of the nodules were adjusted until an agreement was achieved between the investigators. The 12 labeling files and 12 CT images were saved in Dicom format in separate directories and renamed according to the patient identification numbers. Finally, images were trimmed to a size of 96^*^96 pixels placing the nodules in the center by OpenCV 4.2.0 based on Python 3.7. The entire procedure is shown in Figure 2.

In the deep learning procedure, a code name that can be recognized by the machine is required to represent different classes of data. In our research, Class 0 and 1 were chosen for their simplicity. All the nodules in the AIS and MIA stages were marked with Class 0, and all the nodules in the IA stage (including the MPs) with Class 1. All the indices were recorded in a CSV file. In the further task to predict the MPs, a third group exclusively for images labeled MPs were built and named Class 2. The grouping process facilitated the recognition of images by the deep learning models in an organized manner.

### Model Architecture

The invention of the classic LeNet model in 1998 was regarded as the beginning of deep learning (20). Since the AlexNet was reported in 2012, there have been brilliant development of the convolutional neural network (CNN). Many outstanding network structures have been proposed, including the VGG net in 2014 that deepened the model structure, and the ResNet in 2015 that utilized the residual learning methods to process the degradation of the deep network structure. The DenseNet in 2018 enhanced the reuse of the feature map (21–23).

There are several basic structures of the CNN model. The convolutional layer convolves the input parameter and assists with processing images so that they are abstracted to a feature map (24). The pooling layer is used to streamline the underlying computation and reduce the dimensions of the input data (25). The fully connected layer is analyzed with a flattened input matrix to classify the images.

**Figure 3:**
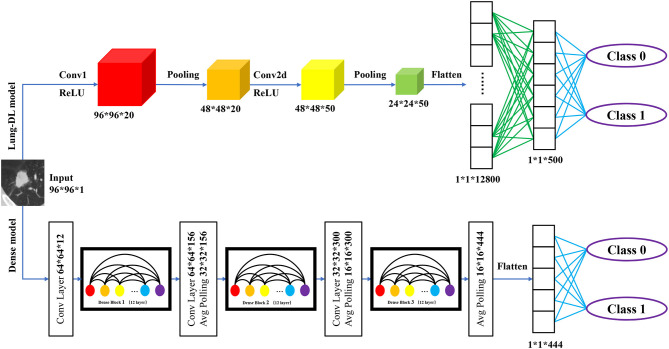
Structure of two deep learning model structure. The Lung-DL model on the top of the picture consists of two convolutional layers, each followed by an average pooling layer. Two fully connected layers were attached to the end of the network; The Dense model on the bottom consists of three dense blocks. Each block consists of 12 convolutional layers. A fully connected layer was attached to the end of the model.

In this research, we chose adapted DenseNet and LeNet, with additional details listed below. The entire structure is shown in Figure 3. The research was performed with an Nvidia RTX 2070 Super graphics processing unit (GPU). Our models were developed with Python 3.7 and Keras 2.3.1 on an Ubuntu 18.04 platform.

#### Lung-DL Model

The first model, which is called the Lung Deep Learning model (Lung-DL model), was adapted from the LeNet model. The model consists of two convolutional layers each followed by an average pooling layer (20). The ReLU function was chosen to be the activation function. Two fully connected layers were attached to the end of the network.

#### Dense Model

The second model was adapted from the DenseNet model. The most unique feature of the DenseNet is its dense block that enhances the reuse of feature maps. As Gao Huang et al. demonstrated in 2018, the layers in the block will receive the feature-maps of all preceding layers. The layers between dense blocks are referred to as transition layers and change feature-map sizes via convolution and pooling (22).

In our model, three dense blocks were used. Each block consisted of 12 convolutional layers. The adjacent two dense blocks were attached by a convolutional layer and an average pooling layer. A fully connected layer was attached to the end of the model.

### Statistical Analysis

In our research, some data was shown in the form of number (percentage), the other data were expressed as mean ± standard deviation. Receiver operating characteristic (ROC) curves were applied to evaluate the two-class classification models using the machine learning module scikit-learn 0.22.1 basing on Python3.7 (26).

## Results

### Dataset Characteristics

In our research, a dataset of 291 patients was established. Table 1 shows the baseline data for the patients. There are 61 (20.96%) AISs, 80 (27.49%) MIAs, and 150 (42.96%) IAs. Among the nodules classified as IA, 33 (11.34%) nodules were micropapillary-predominant lung adenocarcinoma (MPs). The age distribution of the patients is 56.52 years ± 10.56 (mean ± standard deviation). With a total of 176 (60.48%) female patients. The diameters of 104 (35.74%) nodules were <1 cm, while the remainder 187 (64.26%) nodules were larger than 1 cm.

**Table 1:** The baseline of the patients included in the dataset.

	**Train and validation**	**Test**	**Total**
	**(*n* = 241)**	**(*n* = 50)**	**(*n* = 291)**
**Pathologic stage**			
AIS	47 (19.50%)	14 (28.00%)	61 (20.96%)
MIA	69 (28.63%)	11 (22.00%)	80 (27.49%)
IA	97 (40.25%)	20 (40.00%)	117 (40.21%)
MPs	28 (11.62%)	5 (10.00%)	33 (11.34%)
Age (y) (mean ± std)	56.52 ± 10.29	58.04 ± 11.80	56.78 ± 10.56
**Sex**			
Male	98 (40.66%)	17 (34.00%)	115 (39.52%)
Female	143 (59.34%)	33 (66.00%)	176 (60.48%)
**Nodule size**			
(0–1.0 cm]	86 (35.68%)	18 (36.00%)	104 (35.74%)
(1.0–2.0 cm]	124 (51.45%)	26 (52.00%)	150 (51.55%)
(2.0–3.0 cm]	31 (12.86%)	6 (12.00%)	37 (12.71%)
**Nodule location**			
RU	90 (37.34%)	17 (34.00%)	107 (36.77%)
RM	22 (9.13%)	2 (4.00%)	24 (8.25%)
RD	38 (15.77%)	9 (18.00%)	47 (16.15%)
LU	62 (25.73%)	11 (22.00%)	73 (25.09%)
LD	29 (12.03%)	11 (22.00%)	40 (13.75%)

*AIS, adenocarcinoma in situ; MIA, minimally invasive adenocarcinoma; IA, invasive adenocarcinoma; MPs, invasive adenocarcinoma with micropapilary components; RU, the right-up lobe; RM, the right-middle lobe; RD, the right-down lobe; LU, the left-up lobe; LD, the left-down lobe*.

### Patient Tests

**Figure 4:**
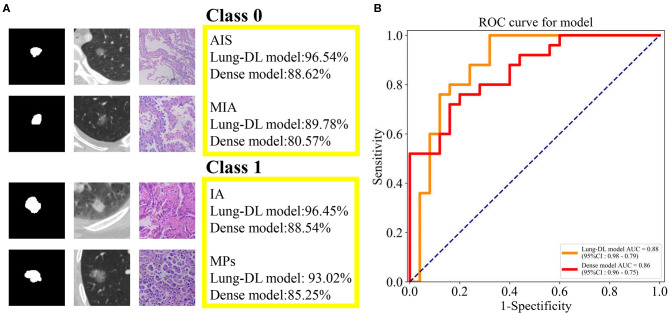
**(A)** The prediction generated by our two models and the pathologic pattern of the nodule examples. **(B)** ROC curve generated from the Lung-DL model and the Dense model in the task to distinguish pathologic invasiveness degree. The Lung-DL model yielded an AUC value of 0.88, and the AUC value of the Dense model was 0.86.

As was illustrated in the data preprocessing, each nodule yielded 12 slices for the test. With the purpose of importing as much information as possible into the model, we aimed to use all 12 slices to obtain the prediction. Therefore, the total prediction percentage was the average value of 12 slices. Examples are shown in Figure 4A. The two classes used is Class 0 for AIS and MIA, and Class 1 for IA and MPs.

For the Lung-DL model, the total result of the test set was 89.52% (Class 0–87.08%, Class 1–91.17%). For the Dense model, the total result of the test set was 81.85% (Class 0–78.44%, Class 1–85.19%). The receiver operating characteristic (ROC) curves generated by the two models were compared in the same figure. The Lung-DL model yielded an AUC value of 0.88, and the AUC value of the Dense model was 0.86, which are shown in Figure 4B.

### Performance of the Model

In this research, the cross-entropy loss function was chosen to accomplish the training task. Every epoch of the training session consisted of a training step and a validation step. Both the Lung-DL model and the Dense model were trained and validated epoch by epoch. When no further improvement was observed in the performance of the model, the training session was automatically terminated. The reduction in the value of loss function is used to evaluate the training quality of the model.

For the Lung-DL model, the termination occurred at the 104th epoch. The validation loss decreased from 0.61 to 0.29, and the validation accuracy increased from 0.66 to 0.87. For the Dense model, the training session terminated at the 94th epoch. The validation loss decreased from 0.79 to 0.30, and the validation accuracy increased from 0.49 to 0.92. A comparison of the two models is listed in Table 2, and the loss function curve and accuracy curve are shown in Supplementary Figure 1.

**Table 2:** The performance comparison of different models.

**No. of classes**	**Model**	**Validation loss**		**Test accuracy**
		**Start**	**End**	
**2**	Lung-DL model	0.61	0.29	89.52%
	Dense model	0.79	0.3	81.85%
**3**	Lung-DL model	0.91	0.34	92%
	Dense model	1.15	0.36	72.91%

*The 2-Class classification taskis used to distinguish the invasiveness degree. The 3-Class classification task is used to predict the micropapillary component*.

### Classification of the Micropapillary-Predominant Nodule (MPs)

As previous researches demonstrated, micropapillary-predominant adenocarcinoma (MPs) has a poorer prognosis than the other four subtypes (4, 6). Based on this statement, we attempted to distinguish the MPs from IA. The code name of the MPs was adapted to Class 2, and three classes were used in this task: Class 0 for AIS and MIA, Class 1 for IA, and Class 2 for MPs. We also explored the ability of our models to classify MPs from other IA nodules.

For the Lung-DL model, the training session terminated at the 131st epoch. The validation loss value decreased from 0.90 to 0.34, and the validation accuracy increased from 0.62 to 0.86. The overall accuracy of the test set was 92% (Class 0–91.18%, Class 1–92.27%, Class 2–95.0%). For the Dense model, the termination occurred at the 106th epoch. The validation loss value decreased from 1.15 to 0.36; the validation accuracy increased from 0.39 to 0.93, and the overall accuracy of the test set was 72.91% (Class 0–73.27%, Class 1–74.24%, Class 2–73.77%). A comparison of different models is listed in Table 2, and the loss function curve and accuracy curve are shown in Supplementary Figure 2.

## Discussion

In our study, we first built a dataset containing pathologic information for 291 lung nodules. Two models adapted from the LeNet and the DenseNet architecture were used to distinguish the AIS and MIA from the IA. Next, knowing that the pathologic subtype of the nodule can assist in guiding resection, we adapted the two models so that they would detect the IA with MPs. We also assessed the performance of the two deep learning models.

After the construction of our models, we focused on two clinical problems. The first problem is that the classification of MIA and IA through pathological biopsy during surgery has a 15.6% possibility of being discordant with the final pathology. The misrecognition of the pathologic invasiveness stage can result in an inappropriate resection range. Insufficient resection range for IA will result in a high risk of locoregional recurrence, and thus, lobectomy is a more optimal surgical approach. On the contrary, given that the 5-year survival after resection of MIA and AIS can reach 100% regardless of the surgery performed, a sublobar resection with a smaller margin is recommended (8). The misrecognition of the pathologic invasiveness stage can result in a second operation or unnecessary excision of lung tissue. In our research, a value of more than 0.85 was obtained for the Lung-DL and Dense models, indicating an ability to thoroughly distinguish the degree of invasiveness. Recognition of the nodule invasiveness stage can guide surgeons to formulate more optimal resection strategies. The issues described above can be avoided if this information can be used to assist medical participators, and thereby increase the efficiency of the medical procedure.

Another problem is that many researchers are demonstrating that a poor prognosis is associated with MPs (4), but there are few approaches available to determine the pathologic subtype. Surgeons are informed of the exact subtype only upon obtaining the final pathology report after resection. As Tsao et al. reported, it is predicted that patients with MPs will benefit from adjuvant chemotherapy (27). If there is a method to determine the pathologic subtype before surgery, a prophylactic plan for an appropriate resection margin and an empirical therapy can be obtained prior to surgery to improve the prognosis for the patients (6). As our research illustrated, an accuracy of more than 70% can be obtained with the two models. Although the imbalance scale of the three classes restricted the performance of models, the result still could reveal the potential to detect the specific pathologic component.

Second, we built a dataset containing the pathologic information of the patients. Due to the essential role of large standard datasets in deep learning, enormous datasets such as LIDC-IDRI (28) have been constructed for public usage. However, only a handful of them contain pathological information that is attached to radiological images. In 2019, Gong et al. collected 828 ground-glass nodules and constructed a dataset (15). Compared to the 1,018 patients with 243,958 slices in LIDC-IDRI, the amount of data containing pathologic information is still not abundant. In our research, we proposed to compensate for the shortage of existing data. Furthermore, we tried to input several slices of nodules into the model, not just merely three slices in three different axes as Gong et al. illustrated in their research (15).

Third, we proposed two models built with the CNN architecture. Since the invention of LeNet-5 in 1998, profound development has occurred in deep learning. Many models emerged after the design of the AlexNet in 2012. In the medical field, the deep learning method has been applied to lesion segmentation, detection, and malignancy classification. The two models presented in our research revealed abilities to classify the invasiveness degree and the pathologic subtype of lung adenocarcinoma. The utility of deep learning techniques in clinical diagnosis procedures can assist surgeons in enhancing the accuracy of diagnosis and supporting precise individualized treatment plans.

We also compared the performance of the two models. According to our research, the Lung-DL model generally outperformed the Dense model due to its fast training speed and more optimal performance, which partly arose from its simpler structure. The Dense model was rather complicated in structure and was overwhelmed with unnecessary information for solving a simple, two-class classification task. It was also noteworthy that the reusing of features, a characteristic function of the Dense model, backfired and led to more mismatching and a less satisfactory outcome.

Several limitations remain to be addressed in our research. First, data insufficiency persisted and could lead to bias during the training session. The dataset scale limited the performance of the model, and the advantage based on a large dataset has not been rigorously proved. The insufficiency of data resulted in an unsatisfactory performance when generating feature maps. As Song et al. combined imaging parameters with clinical features to identify pathologic components (6), if the clinical features manually labeled in a radiomic fashion can be used in our labeling procedure as a complement, more information can be sent to the fully connected layers at the end of the model, which will increase the performance and stability of the model. Second, in our research, we did not introduce an external dataset for validation, partly because of the lack of a standard public lung nodule dataset that contained pathologic information. The performance of the model still requires validation in another cohort. A comparison between radiologists and AI models is also a method that can be used to validate the practicability of using deep learning models in the clinical procedure. Last but not least, because of the limitation of resources, we can only conduct single-center research, which restricted the performance of the models and the application of the research has not been dug completely.

Further research would involve the introduction of radiomic methods into deep learning models as radiomic methods readily expand the required datasets and features and receive augmentation in the upper limit of accuracy and stability from deep learning models. Another possibility is the conduction of malignancy prediction using a combination of AI extracted features and handcrafted features. Further applications will be explored when more initial studies in this field are come up.

## Conclusion

Herein, we proposed two deep learning models based on the LeNet and DenseNet to generate predictions. We evaluated their usefulness in the prediction of invasiveness of lung adenocarcinoma along with their capability to discriminate MPs from other subtypes. The results showed that deep learning models can distinguish different subtypes of lung adenocarcinoma and can detect certain pathologic components. Thus, our models can assist radiologists to better distinguish the invasiveness degree of lung nodules and help surgeons to make their operation choice more appropriately.

## Data Availability Statement

The original contributions presented in the study are included in the article/Supplementary Material, further inquiries can be directed to the corresponding author/s.

## Ethics Statement

The studies involving human participants were reviewed and approved by the Institutional Review Board of Jiangsu Cancer Hospital and Jiangsu Institute of Cancer Research. The patients/participants provided their written informed consent to participate in this study. Written informed consent was obtained from the individual(s) for the publication of any potentially identifiable images or data included in this article.

## Author Contributions

HD and WX performed the deep learning model, analyzed the data, and wrote original draft preparation. LZ collected the raw CT image data. BC and YZ labeled the image data and built the dataset. QM and BC reviewed and edited the manuscript. LX, FJ, and GD designed the study, provided insights on methodology, data interpretation, and manuscript review and editing. All authors contributed to the article and approved the submitted version.

## Conflict of Interest

The authors declare that the research was conducted in the absence of any commercial or financial relationships that could be construed as a potential conflict of interest.

## Supplementary Material

Supplementary Figure 1 The loss function curve and accuracy curve for two models in the 2-Class classification task.

Supplementary Figure 2 The loss function curve and accuracy curve for two models in the 3-Class classification task.
