# Supplementary figures and images for: CT-Based Deep Learning Model for Invasiveness Classification and Micropapillary Pattern Prediction Within Lung Adenocarcinoma
Source: Front Oncol. 2020 Jul 22;10:1186. doi: 10.3389/fonc.2020.01186 (PMC7388896; doi:10.3389/fonc.2020.01186)

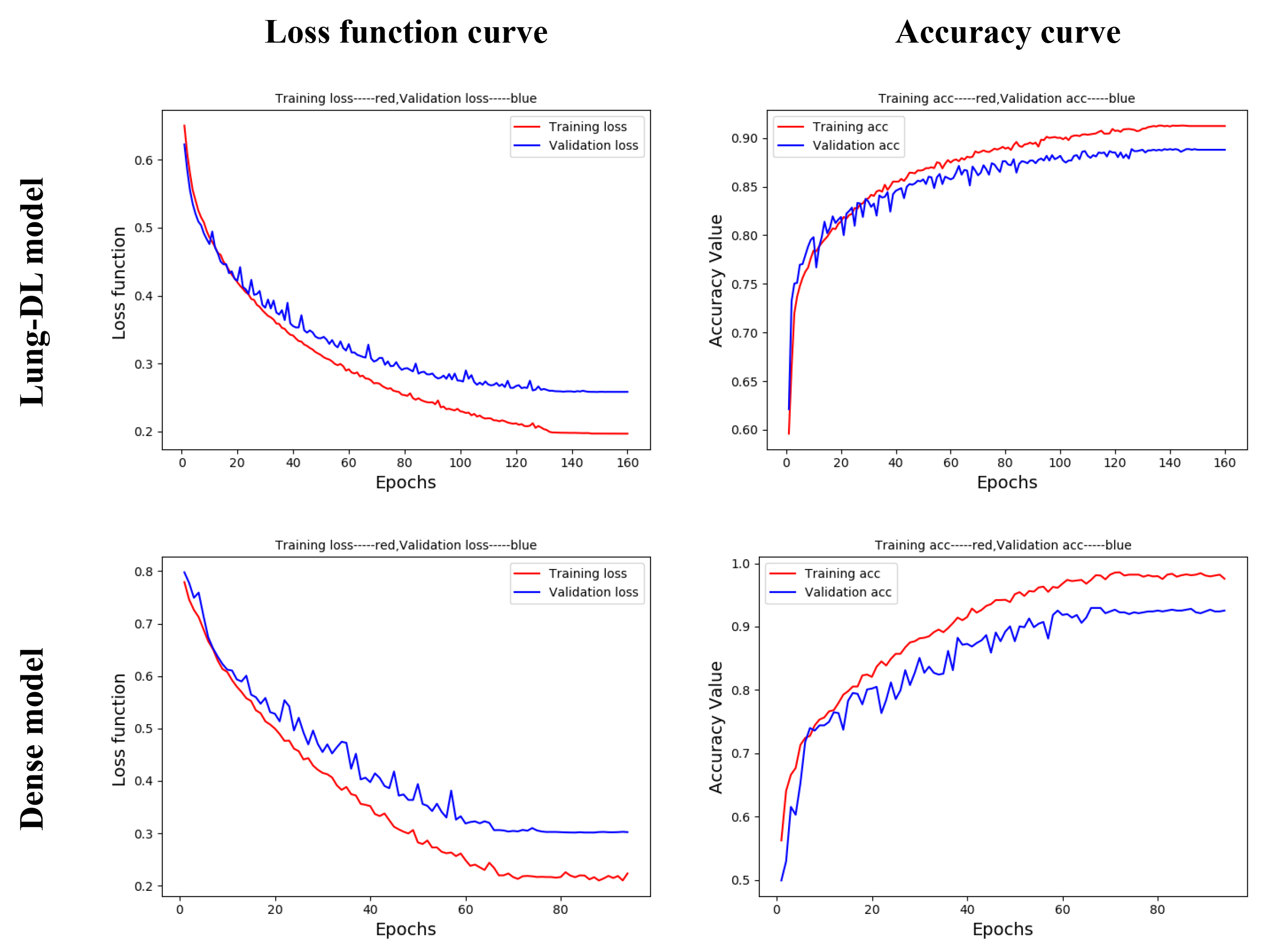

Supplement: Supplementary Figure 1 — The loss function curve and accuracy curve for two models in the 2-Class classification task. [file Image_1.TIF]

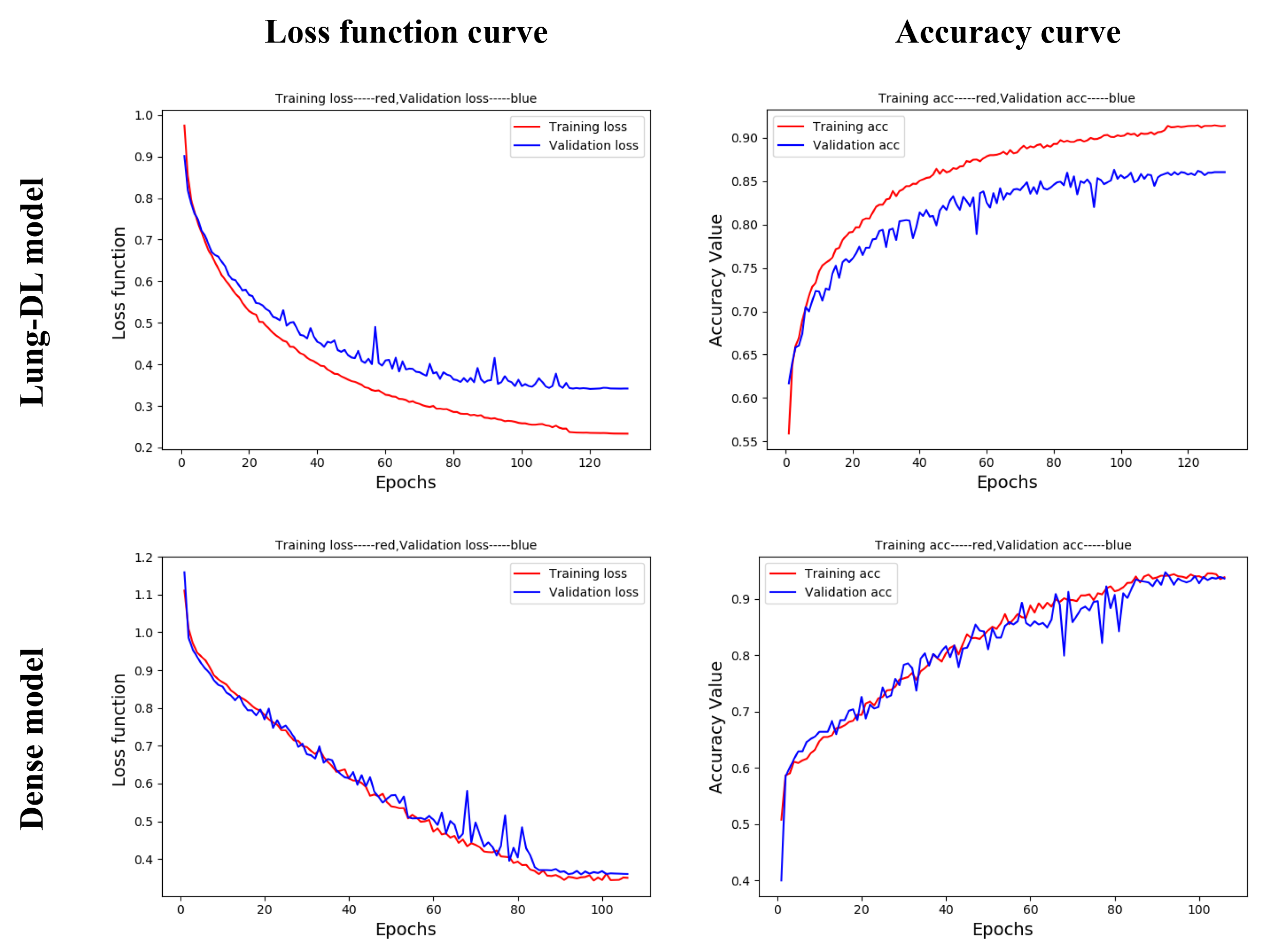

Supplement: Supplementary Figure 2 — The loss function curve and accuracy curve for two models in the 3-Class classification task. [file Image_2.TIF]
